# Effect of tumor microenvironment on ferroptosis: inhibition or promotion

**DOI:** 10.3389/fonc.2023.1155511

**Published:** 2023-05-05

**Authors:** Zhengzhen Xia, Yi Quan

**Affiliations:** ^1^ The First Clinical Medical School, Guangdong Medical University, Zhanjiang, Guangdong, China; ^2^ Department of Oncology Medical Center, The First People’s Hospital of Zhaoqing, Zhaoqing, Guangdong, China

**Keywords:** ferroptosis, tumor microenvironment, tumor immunotherapy, lipid peroxidation, micro RNA

## Abstract

Ferroptosis is a type of lipid peroxidation-induced, iron-dependent programmed cell death. Emerging evidence suggests that ferroptosis is intimately connected to tumorigenesis, development, treatment and plays a major role in tumor immune regulation. This study focused on the connection between ferroptosis and immune regulation, which may offer a theoretical basis for targeting ferroptosis and tumor immunotherapy.

## 1 Introduction

Unlike apoptosis, different types of necrosis, and autophagy, ferroptosis is a recently identified type of programmed cell death that is regulated by iron-dependent lethal lipid peroxidation (1). Ferroptosis mainly occurs as a result of reduced biological activity of glutathione peroxidase 4 (GPX4), lipid peroxidation, and rise of reactive oxygen species (ROS). A crucial factor in the occurrence of ferroptosis is GPX4. GPX4 inhibits ferroptosis by reducing ROS to glutathione (GSH) (2). The cystine/glutamate antibody (system xc-) made up of light chain subunit SLC7A11 and heavy chain subunit SLC3A2, which exchange cystine and glutamate in equal amounts. The ingested cystine undergoes a series of reactions to synthesize glutathione and maintain GPX4 activity. Tumor immunotherapy is a prospective method to address the treatment of malignancies in clinical practice, but only a small proportion of patients are benefited from this approach (3). The tumor microenvironment (TME) also has a significant role in treatment outcome. TME is a hypoxic, acidified, inflammatory, and immunosuppressive state. TME contains a considerable number of immune cells, such as effector CD8 T cells with antitumor effects, natural killer (NK) cells, dendritic cells (DCs), and regulatory T cells (Tregs) with immunosuppressive functions, as well as myeloid-derived suppressor cells (MDSCs), tumor-associated macrophages (TAMs), tumor-associated neutrophils (TANs), and cancer-associated fibroblasts cells (CAFs). The combination of immunotherapy with other therapeutic modalities reverses the immunosuppressive state and has a synergistic antitumor effect (4). In this case, targeted ferroptosis combined with immunotherapy is a viable therapeutic tool. This review describes the interaction between ferroptosis and tumor immunotherapy in order to provide a theoretical reference for the combination of targeted ferroptosis with immunotherapy.

### 1.1 Characteristics of TME

#### 1.1.1 Hypoxia

Hypoxia is a common phenomenon in solid tumors, wherein chronic hypoxia promotes tumor growth, metastasis (5), and therapy resistance (6) by remodeling the microenvironment. The stabilization of hypoxia-induced hypoxia-inducible factors (including HIF1α, HIF2α, and HIF-3α) activate hypoxia target genes thereby regulating angiogenesis, cell growth, glycolysis, DNA damage repair, and even apoptosis (7). Under hypoxia, HIF regulates CD8 T cell infiltration and activity *via* von Hippel-Lindau (VHL) (8). HIF-1α is the key promoter of immunosuppressive TME (9). Hypoxic TME inhibits the activation of cytotoxic T lymphocytes (CTLs), reduces interferon-gamma (IFNγ) production and release, promotes Treg activity (10, 11), promotes the differentiation of MDSCs to immunosuppressive TAMs to enhance immunosuppression (12), impairs DC (13) and NK cell (14, 15) activation and function, and induces epithelial to mesenchymal transition (EMT) (16, 17). In addition, hypoxia regulates the levels of various microRNAs (miRNAs) and long non-coding RNAs (IncRNAs), and these ncRNAs influence the activation of HIF-1α and consequently create positive or negative feedbacks to regulate hypoxic TME (18–21).

#### 1.1.2 Acidification (lactate)

The weak acidity of TME is caused by the excessive consumption of glucose by cancer cells, resulting in lactate accumulation. The Warburg effect states that the energy of cancer cells is dominated by glycolysis even under oxygen-sufficient conditions, producing large amounts of lactate (22). The creation of lactate impairs the CTLs’ capacity to multiply and produce cytokines, which reduces their cytotoxic activity (23), suppresses the production of IFNγ by NK and NKT cells, and reduces their antitumor function (24, 25), mediates the differentiation of DCs (26), increases Treg activity (27), and recruits macrophages and induces their functional polarization into TAMs (28), leading to immunosuppression and promoting tumor cell growth and metastasis.

#### 1.1.3 Cancer-related inflammation

Strikingly, cancer-related inflammation is caused by proinflammatory cytokines and chemokines produced by tumor cells, resulting from cancer gene-mediated malignant transformation (29). Chronic inflammation has been proven to be among the primary causes of tumor occurrence and growth (30). Macrophages and lymphocytes are the primary immune cells that infiltrate sites of persistent inflammation, which can indirectly or directly promote tumor development by releasing various cytokines. These cytokines are categorized into proinflammatory cytokines [interleukin (IL)-1, IL-6, IL-15, IL-17, IL-23, tumor necrosis factor-alpha (TNF-α), and IFN-γ] and anti-inflammatory cytokines [IL-4, IL-10, IL-13, and tumor growth factor-beta (TGF-β)] (31). Inflammation is an effective inducer of EMT in tumors, TGF- β, TNF-α, IL‐1β, IL-6, IL-8, and other proinflammatory cytokines play a role in the initiation and maintenance of tumor EMT, increasing the invasiveness of cancer cells and promoting tumor metastasis (32–35). Another mechanism of cancer-related inflammation involves the emergence of damage-associated molecular patterns (DAMPs). Various endogenous ligands of DAMPs are produced in reacting to hypoxia, cell stress, and tissue damage and then detected by pattern recognition receptors (PRRs) generated by innate immune cells. These DAMPs include high-mobility group box-1 (HMGB1), heat shock protein, S100 protein, and ATP (36, 37).

### 1.2 Effect of TME on ferroptosis

#### 1.2.1 Hypoxia with ferroptosis

Hypoxia upregulates iron regulatory protein 2 (IRP2) through a post-translational mechanism to increase iron transport (TFRC) and SLC11A2 while decreasing iron storage (FTH) (38). A vital cellular regulator of tumor hypoxia, carbonic anhydrase IX (CAIX), controls intracellular and extracellular pH and acidosis (39). CAIX prevents cancer cells from undergoing iron death *via* the cystine/glutamate antiporter xCT (40). A previous study has shown that hypoxia protects cancer cells from iron death through multiple pathways. Hypoxia-induced HIF-1α/lncRNA-PMAN prevents ferroptosis in gastric cancer by enhancing SLC7A11 mRNA stability and increasing the expression of SLC7A11 at the posttranscriptional level (41). The CBSLR/CBS signal axis regulates the methylation of ACSL4 to prevent ferroptosis in gastric cancer by polyubiquitination (42). Blocking ferroptosis of hepatocellular carcinoma by inhibiting METTL14 triggered YTHDF2-dependent silencing of SLC7A11 (43). In addition, hypoxia reduces NCOA4 in macrophages, thereby increasing FTMT and FTH and preventing ferroptosis (44).

Recently, Zhao et al. designed a dual hypoxia-sensitive polymeric nanocarrier containing azobenzene and nitroimidazole, encapsulating the small molecule ferroptosis inducer RSL3, and validated it in a nude mouse model of ovarian cancer. The study also demonstrated that the polymeric carriers depleted NADPH in tumor cells under hypoxic conditions, leading to depletion of GSH and Trx and further inhibition of GPX4 activity, thereby sensitizing tumor cells to ferroptosis. Furthermore, it functions like a selective ferroptosis sensitizer solely in hypoxic tumors, without damaging healthy organs (45). This study provides a novel idea for efficacy enhancement and toxicity reduction of ferroptosis antitumor therapy.

#### 1.2.2 Lactate with ferroptosis

The rapid proliferation of cancer cells relies on glycolysis as an energy source, producing a large amount of lactic acid. The lactic acid in the microenvironment provides energy for cell growth and development, affects the biochemical function of proteins in cells as a vital signaling molecule, regulates the biological functions of various cells, and promotes the malignant proliferation and progress of tumors (46). Luo et al. confirmed that lactic acid inhibits the ferroptosis of hepatoma cells through *in vivo* and *in vitro* experiments. Excessive lactic acid upregulated the expression of hydroxycarboxylic acid receptor 1 (HCAR1) and monocarboxylate transporter 1 (MCT1) and inhibited ferroptosis of liver cancer cells by reducing the lipid peroxidation of liver cancer. Strikingly, the inhibition of HCAR1/MCT1 increases the proportion of AMP/ATP in hepatoma cells, enhances the AMP-activated protein kinase’s phosphorylation, downregulates the level of mature of sterol regulatory element-binding protein 1 and its target gene stearoyl-coenzyme A (*CoA*) desaturase-1, and enhances the ferroptosis sensitivity of hepatoma cells (47). This finding suggested that additional studies on the mechanism of lactic acid and ferroptosis would identify the drug targets for tumor treatment and improve the prognosis of patients.

#### 1.2.3 Cancer-related inflammation with ferroptosis

Proinflammatory factors in the TME may promote or inhibit tumor initiation and progression by affecting tumor ferroptosis. In head and neck squamous cell cancer, IL-6 inhibits ferroptosis by transcriptionally increasing xCT expression through the JAK2/STAT3 pathway (48). miR-539 directly triggers the SAPK/JNK protein kinase through targeted TNF-α-induced protein 8 to promote colorectal cancer ferroptosis (49). CircABCB10 silencing promotes rectum cancer cell ferroptosis and apoptosis through targeting the miR-326/CCL5 axis (50). Circ-IL-4 receptor controls the miR-541-3p/GPX4 axis to inhibit ferroptosis and promote carcinogenesis in hepatocellular carcinoma (51). HMGB1 regulates acute myeloid leukemia cell ferroptosis through the RAS-JNK/p38 pathway (52).

### 1.3 Ferroptosis with immune effector cells

#### 1.3.1 Ferroptosis with CD8 T cells

The main agents of antitumor immunity, CD8 T cells secrete IL-2, IL-12, and (IFNγ) in TME, which increases the ability of CD8 T cells to target and destroy tumor cells. Interestingly, IFNγ produced by immunotherapy-activated CD8 T cells suppresses the expression of SLC3A2 and SLC7A11, two components of the glutamate-cystine anti-transport protein system xc-, and prevents tumor cells from utilizing cystine, promoting lipid peroxidation and ferroptosis in tumor cells (53, 54). Another study found that IFNγ generated in the TME by activated T cells paired with arachidonic acid affected spontaneous antitumor immunity *in vivo* by inducing tumor cell ferroptosis through acyl-CoA synthetase long-chain family member 4 (ACSL4) (55). These studies highlighted that targeting the metabolism associated with tumor ferroptosis can improve the efficacy of cancer immunotherapy.

**Figure 1:**
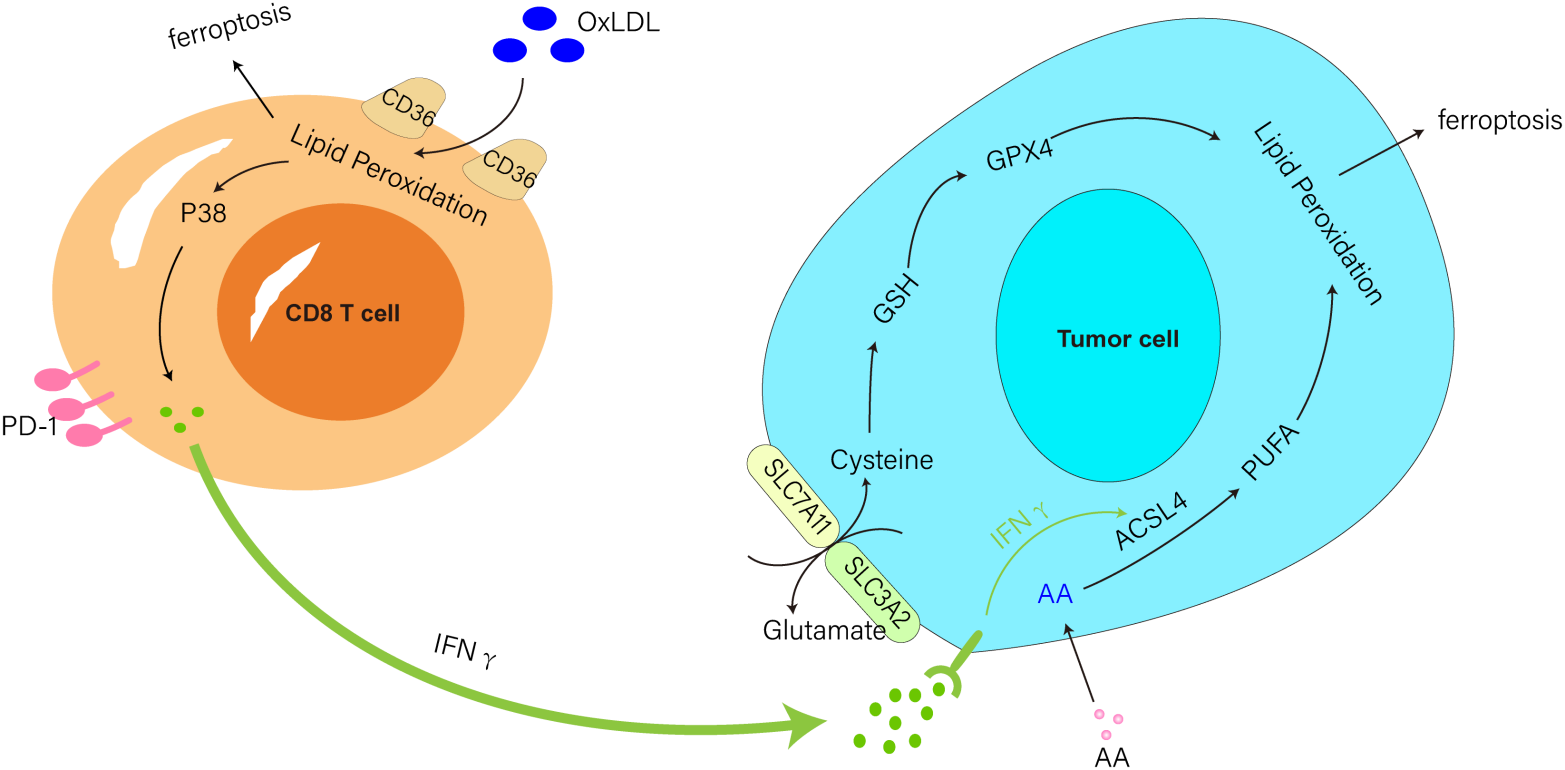
IFNγ produced from CD8 T cells triggered by immunotherapy downregulated SLC3A2 and SLC7A11 activity and aided ferroptosis in tumor cells. IFNγ produced by activated T cells paired with AA-induced tumor cell ferroptosis *via* ACSL4. CD36 mediated the uptake of oxLDL by CD8 T cells to induce ferroptosis and P38 phosphorylation and inhibited IFNγ production in T cells.

Recently, Ma et al. found that oxLDL in the TME induced iron death and p38 phosphorylation by lipid peroxidation in CD8+ T cells in a CD36-dependent manner. Also, the activation of P38 induced CD8 T cell death, suppressed IFNγ and TNFα generation in T cells, depleted CD8 T cells, and reduced antitumor effects (56, 57) (**Figure 1**). In addition, the research team also discovered that anti-PD-1 antibodies and CD36 deletion in CD8 T cells together showed superior antitumor effects to either treatment alone (57). This research suggested that targeting CD36 and ferroptosis might be a viable option for improving the antitumor efficacy of T-cell based immunotherapy.

#### 1.3.2 Ferroptosis with NK cells

NK cells are nature leukomonocytes that are essential for tumor surveillance and control and in tumor immunotherapy due to their ability to kill abnormal cells without any antigenic stimulation and persistent immune memory. NK cells trigger stressed cell death primarily by the release of perforin, granzyme, and other lytic granule molecules (58). In addition, NK cells have a cytotoxic capacity similar to that of CD8 T cells, regulating immunity and killing tumor cells by releasing IFN-γ (59). Another study demonstrated that activating mitochondrial apoptosis in cancer cells enhances NK cell death. Thus, initiating mitochondrial apoptosis *via* BH3 mimics can synergize with NK cells to kill cancer cells (60). Moreover, the synergistic effect of BH3 mimetic drugs in combination with ferroptosis induces cancer cell death (61). Then, targeted ferroptosis combined with NK cell therapy may also be a viable strategy. Kim et al. found that the use of clinical-grade iron oxide nanoparticles (ferumoxytol) to mediate ferroptosis in prostate cancer activated NK cells and increased the cytotoxic function of NK cells. Furthermore, in the therapy of ferroptosis+NK cells, tumor cells expressed HMGB1 and PD-L1, and a clear regression of tumor volume after ferumol-mediated ferroptosis and NK cell treatment was observed in a prostate cancer mouse model (62). These findings suggested that ferroptosis can improve NK cell activity.

#### 1.3.3 Ferroptosis with DCs

DCs are robust antigen-presenting cells (APCs) in the organism that promote immunization or tolerance by sampling and presenting antigens to T cells as well as transmitting immunomodulatory signals *via* cell-cell interaction and cytokines. promoting immunity or tolerance by sampling and presenting antigens to T cells and delivering immunomodulatory signals through cell-cell contact and cytokines (63). DCs consist of exogenous antigens on major histocompatibility class I (MHC-I) molecules and initiate CD8 T-cell antitumor immunity *via* antigen cross-presentation (64). The activation of DCs can be achieved by PRRs on DCs that recognize pathogen-associated molecular patterns (PAMPs) or DAMPs (65). However, a new research study found that although ferroptosis is capable of releasing cytokines and DAMPs, it does not activate antitumor immune responses, inhibits cross-presentation of soluble antigens to DCs, impairs DC maturation, and inhibits phagocytosis of tumor cells by DCs (66). According to this phenomenon, cancer cells’ ferroptosis may not represent an immunological type of cell death.

### 1.4 Ferroptosis with immunosuppressive cells

#### 1.4.1 Ferroptosis with tregs

Tregs are essential for maintaining self-tolerance and immune cell homeostasis, and their mediated immunosuppression is a major obstacle to tumor immunotherapy. Tregs exhibit their immunosuppressive function through various mechanisms, including inhibition of APCs *via* cytotoxic T lymphocyte antigen-4 (CTLA-4), suppression of cytokine secretion (IL-10, TGF-β, and IL-35), and competitive depletion of IL-2. Oxidative stress-induced apoptosis of Treg cells in tumors amplifies their immunosuppressive effects and abolishes the efficacy of PD-L1 blockade therapy (67). A current research revealed that GPX4 prevents lipid peroxidation and ferroptosis in Tregs and that GPX4-deficient Tregs promote mitochondrial superoxide production and IL-1β production, thereby promoting T helper 17 (Th17) responses. Furthermore, GPX4 deficiency, rather than intra-tumor Treg cell apoptosis, enhances antitumor immune function and inhibits tumor growth by inducing ferroptosis (68). The current results indicated that induction of iron death in Treg cells could be an effective strategy to improve cancer therapy.

#### 1.4.2 Ferroptosis with MDSCs

MDSCs constitute a group of immunosuppressive immature bone marrow cells that promotes tumor progression through various non-immune mechanisms to suppress antitumor immunity and support tumor growth and are essential for encouraging tumor immune escape (69). N-Acyl sphingomyelin hydrolase (ASAH2) is overexpressed in MDSCs of colon cancer and protects MDSCs from ferroptosis by regulating p53 stabilization and inhibiting Hmox1 expression from reducing lipid ROS. The novel MDSC-targeting medication NC06 induces MDSC ferroptosis by activating the p53-Hmox1 pathway and decreases MDSC accumulation to stimulate T-cell tumor infiltration and inhibit tumor growth *in vivo* (70). Thus, induction of MDSC ferroptosis is a potentially effective therapy for tumor immunotherapy.

#### 1.4.3 Ferroptosis with TAMs

**Figure 2:**
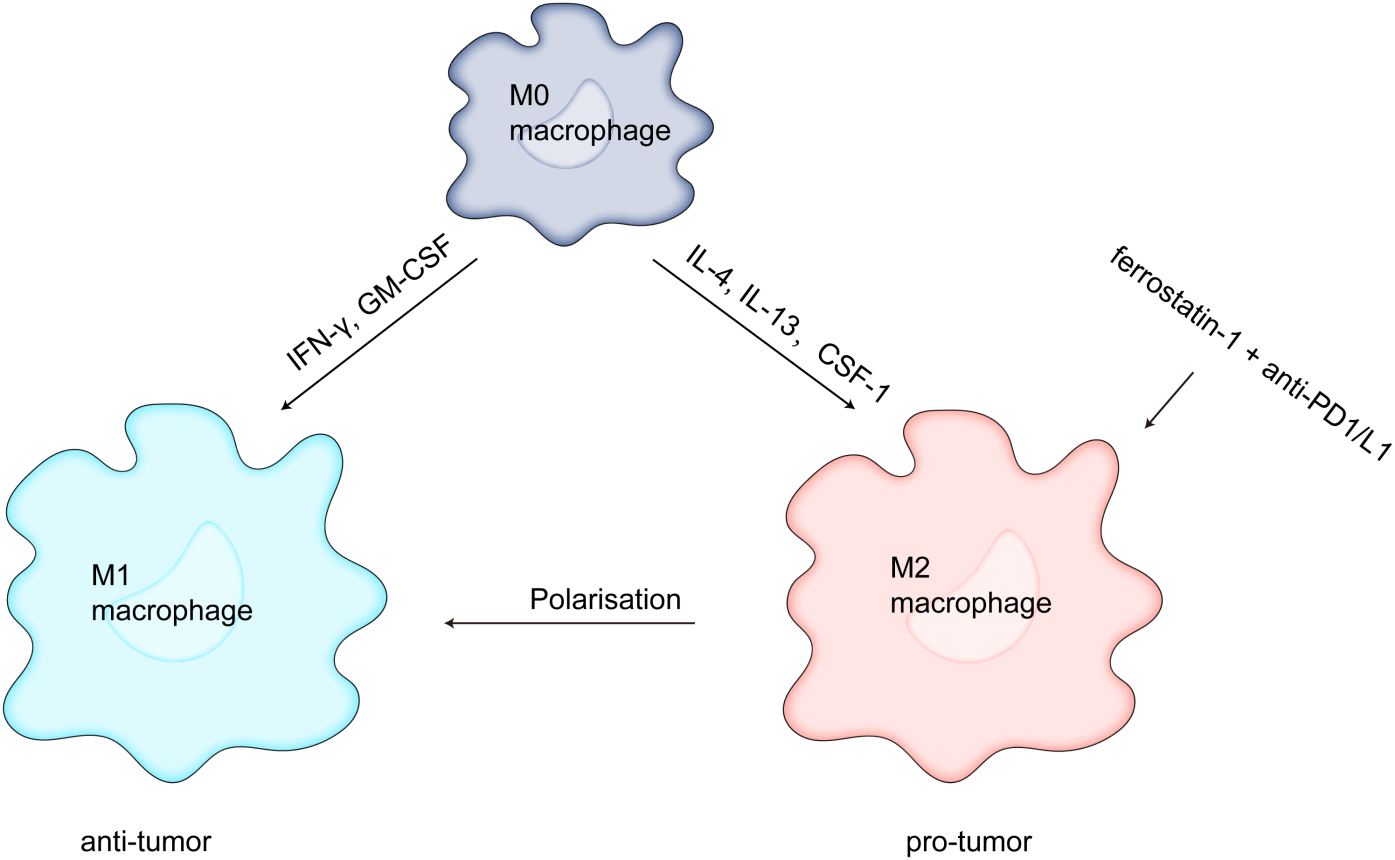
Ferrostatin-1 and anti-PD1/L1 together diminish TAM infiltration and change the M2-like phenotype into the M1-like phenotype, which strengthens the anticancer impact.

Macrophages can be divided into two subtypes based on their function and activation. M1 macrophages (driven by bacterial products and interferons produced during type 1 immune responses activated by type 1 T helper and innate lymphoid cells) kill tumor cells and M2 macrophages (driven by type 2 T helper and innate lymphoid cells, such as IL-4 and IL-13) promote cancer progression and immunosuppression (71). TAMs are widely considered to be M2 macrophages, and the M2-like TAMs prevent CD8 T-cell antitumor effect and DC development by secreting transforming growth factor-beta (TGF-β) and IL-10 (72). Several studies have focused on targeting macrophages, and Wang et al. showed that ZVI-NP selectively triggers ferroptosis in lung cancer cells by inhibiting the NRF2-mediated cytoprotective program, shifts pro-tumor M2 macrophages to antitumor M1, enhances antitumor immunity by increasing the cytotoxic function of CD8 T cells and decreasing the Tregs ratio (73). Gu et al. found that MIL88B/RSL3 nano preparations inhibited metastatic tumor activity by polarizing macrophages from pro-tumor M2 to antitumor M1 phenotypes *via* a ferroptosis-enhanced metabolic program (from mitochondrial oxidative phosphorylation to glycolysis), activated of numerous proinflammatory signals, and greatly inhibited tumor angiogenesis (74). Xu et al. demonstrated that downregulation of matrix remodeling-associated protein 8 (MXRA8) induced ferroptosis in gliomas by elevating Fe levels and attenuating M2 macrophage infiltration to inhibit tumor progression (75). Li et al. found that dihydroartemisinin produced ferroptosis in TAM-induced ROS/LPO, resulting in DNA damage, which in turn activated downstream NF-κB to remodel TAM to M1 phenotype, and that the remodeled M1 macrophages exerted antitumor effects (76). Tang et al. discovered that macrophages with xCT deletion dramatically reduced TAM infiltration, blocked M2-like phenotypic changes in HCC tumor tissue, activated and elevated ferroptosis activity, and hampered tumor growth and metastasis. Moreover, PD-L1 expression in macrophages was markedly elevated by xCT-mediated macrophage ferroptosis, which also enhanced the anti-tumor effectiveness of anti-PD-L1 therapy (77). According to research by Hao et al., blocking APOC1 can encourage M2 macrophages to become M1 macrophages through the iron-sparing pathway, modifying the TME and enhancing the effectiveness of anti-PD1 immunotherapy for hepatocellular carcinoma (78). However, some studies have shown drawbacks in ferroptosis-mediated cancer treatment. The study also demonstrated that, although ferroptosis increases immune cell activation and infiltration, it attenuates the antitumor cytotoxic killing effect. Conversely, TAMs infiltration are reduced and converted TAM to M1-like phenotype when ferrostatin-1 and anti-PD1/L1 antibodies are combined (79) (**Figure 2**). The above results suggested that ferroptosis plays a major role in TAM infiltration and activation in TME.

#### 1.4.4 Ferroptosis with TAN

As the main component of TME, TAN promotes tumor progression and metastasis in communication through a variety of growth factors, chemokines, inflammatory factors, and other immune cells (80). Similar to macrophages, neutrophils can polarize into an antineoplastic (N1) or tumor-promoting (N2) phenotype. N1 TAN exerts antitumor activity through direct or indirect cytotoxicity, while N2 TAN stimulates immunosuppression, tumor growth, angiogenesis, and metastasis through DNA instability or the release of cytokines and chemokines (81). Li et al. demonstrated that tumor lesions occurring during early tumor progression (i.e. ischemia) draw neutrophils to the areas of tissue injury, where neutrophils destroy tumor cells by ferroptosis, resulting in necrosis. In addition, a cycle of positive feedback between necrosis and neutrophil infiltration increases intratumoral necrosis and speeds up glioma mortality (82).

#### 1.4.5 Ferroptosis with CAFs

CAFs are critical components of TME and sources of cytokines, growth factors, and exosomes (83). CAF-secreted exosomes influence tumor phenotypes, whereas exosomes released by tumor cells activate CAFs (84). CAFs derived exosomes transmit biological information *via* miRNAs, lncRNAs, circRNAs, lipids, and proteins and promote tumor growth (85), invasion (86, 87), metastasis (88), and angiogenesis (89), induce EMT (90, 91), endow tumor with chemical (92, 93) and radiation resistance (94), impede the action of tumor-infiltrating immune cells (95), and regulate TME’s antitumor immunological state. A recent study found that CAFs suppress ALOX15 expression by secreting exosomal miR-522, affecting lipid-ROS production in tumors to suppress ferroptosis in gastric cancer cells. In addition, chemotoxicity affects tumor growth and attenuates cisplatin chemotherapeutic efficacy by boosting miR-522 release from CAFs through triggering the USP7/hnRNPA1 pathway, whereas preventing miR-522 secretion could inhibit tumor growth and enhance sensitivity to cisplatin (96). The present study illustrated that CAFs-derived exosomes regulate tumor ferroptosis by transporting special signaling information and triggering cell ferroptosis in tumors by blocking the packaging of specific miRNAs into exosomes could be a viable novel therapeutic approach.

## 2 Prospects

**Figure 3:**
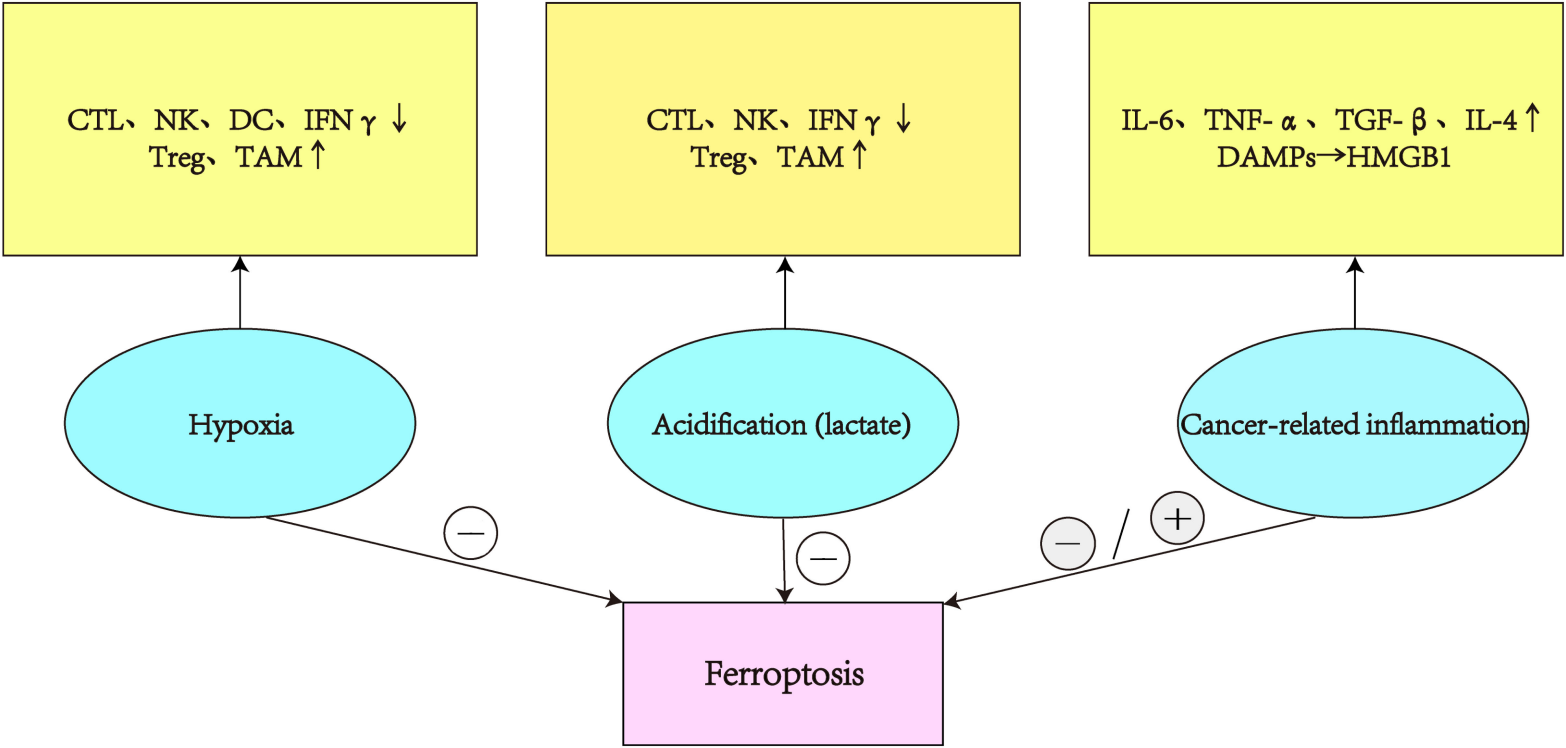
In the TME, hypoxia and acidity reduce the activity of CTL, NK, DC, and INF, boost the immunosuppressive effects of Treg and TAM, and prevent tumor ferroptosis. Chronic inflammation of tumors secretes various cytokines that promote or inhibit tumor ferroptosis.

Immunotherapy is a potential method for treating cancer that achieves tumor clearance through reshaping the TME and activating antitumor immune responses. Current studies with immunotherapy alone have failed to demonstrate significant survival benefits, possibly because of TME’s intricate immunosuppressive nature.The tumor microenvironment inhibits the anti-tumor effects of CTL, NK, and DC due to its hypoxia, acidification, and inflammatory characteristics. It also increases the activity of immunosuppressive cells, puts the tumor microenvironment in an immunosuppressive state, and prevents the ferroptosis of tumor cells(**Figure 3**). Therefore, reversing the immunosuppressive state of the TME is critical to the success of tumor therapy. Ferroptosis can be promoted or inhibited by immune cells that attack the tumor microenvironment, and the identification of ferroptosis offers fresh hope for halting tumor growth. The combination of targeting the TME and ferroptosis enhances the antitumor effect by promoting the activation and presentation of effector T cells and dendritic cells, inhibiting the activity of immunosuppressive cells and relieving the immunosuppressive state of the tumor microenvironment. Although the combination of targeted ferroptosis therapy with immunotherapy may improve efficacy, the potential drug resistance of ferroptosis cannot be ignored. Consequently, a thorough investigation of ferroptosis’ function in the TME is necessary to build a creative strategy for the creation of fresh cancer treatment options

## Author contributions

ZX and YQ provided direction and guidance throughout the preparation of this manuscript. All authors have read and approved the final manuscript.
